# Differentiating gastric schwannoma from gastric stromal tumor (≤5 cm) by histogram analysis based on iodine-based material decomposition images: a preliminary study

**DOI:** 10.3389/fonc.2023.1243300

**Published:** 2023-11-17

**Authors:** Gang Wang, Xianwang Liu, Junlin Zhou

**Affiliations:** ^1^ Department of Radiology, Lanzhou University First Hospital, Lanzhou, China; ^2^ Key Laboratory of Medical Imaging of Gansu Province, Lanzhou, China; ^3^ Department of Radiology, Lanzhou University Second Hospital, Lanzhou, China; ^4^ Gansu International Scientific and Technological Cooperation Base of Medical Imaging Artificial Intelligence, Lanzhou, China

**Keywords:** gastric neoplasms, schwannoma, stromal tumor, iodine-based material decomposition map, histogram analysis

## Abstract

**Objective:**

This study aims to investigate the value of histogram analysis based on iodine-based material decomposition (IMD) images obtained through dual-energy computed tomography (DECT) to differentiate gastric schwannoma (GS) from gastric stromal tumor (GST) (≤5 cm) preoperatively.

**Methods:**

From January 2015 to January 2023, 15 patients with GS and 30 patients with GST (≤5 cm) who underwent biphasic contrast-enhanced scans using DECT were enrolled in this study. For each tumor, we reconstructed IMD images at the arterial phase (AP) and venous phase (VP). Nine histogram parameters were automatically extracted and selected using MaZda software based on the IMD of AP and VP, respectively, including mean, 1st, 10th, 50th, 90th, and 99th percentile of the iodine concentration value (Perc.01, Perc.10, Perc.50, Perc.90, and Perc.99), variance, skewness, and kurtosis. The extracted IMD histogram parameters were compared using the Mann–Whitney *U*-test. The optimal IMD histogram parameters were selected using receiver operating characteristic (ROC) curves.

**Results:**

Among the IMD histogram parameters of AP, the mean, Perc.50, Perc.90, Perc.99, variance, and skewness of the GS group were lower than that of the GST group (all *P* < 0.05). Among the IMD histogram parameters of VP, Perc.90, Perc.99, and the variance of the GS group was lower than those of the GST group (all *P* < 0.05). The ROC analysis showed that Perc.99 (AP) generated the best diagnostic performance with the area under the curve, sensitivity, and specificity being 0.960, 86.67%, and 93.33%, respectively, when using 71.00 as the optimal threshold.

**Conclusion:**

Histogram analysis based on IMD images obtained through DECT holds promise as a valuable tool for the preoperative distinction between GS and GST (≤5 cm).

## Introduction

Gastric schwannoma (GS) and gastric stromal tumor (GST) represent distinct histological subtypes of gastric submucosal mesenchymal tumors (1). GS originates from Schwann cells in the subepithelial Auerbach’s plexusthe and accounts for approximately 0.2% of gastric tumors. The benign biological behavior makes recurrence and metastasis rare. As a result, a good prognosis can be achieved with complete surgical excision, and only follow-up is required for asymptomatic patients (2). GST originates from the interstitial cells of Cajal, accounting for approximately 2.2% of gastric tumors. Their diverse biological behaviors make them more prone to recurrence and metastasis. Therefore, more aggressive treatment methods and follow-up strategies should be adopted, such as in patients with a high risk of GST receiving preoperative targeted therapy with imatinib (3, 4). When distinguishing between GS and GST (>5 cm in size) is relatively straightforward using conventional imaging features, the challenge arises when GST measures ≤5 cm. The similarity in the age of onset, clinical manifestations, and conventional imaging features make it challenging to distinguish the two tumors preoperatively (3, 5–7). Although puncture biopsy offers an effective means of distinguishing GS from GST (≤5 cm), it is an invasive procedure with potential risks, including tumor rupture and metastases in GST (7). Therefore, exploring a noninvasive and quantitative method to effectively differentiate GS and GST (≤5 cm) preoperatively is of great significance for the clinical selection of treatment options.

Recently, dual-energy computed tomography (DECT) has been increasingly used in clinical practice. An iodine-based material decomposition (IMD) image can be generated by DECT based on basic material decomposition technology. The IMD images offer a visual reflection of iodine distribution within tissues and can be quantitatively assessed by iodine concentration measurements (8). Previous studies have shown that the heterogeneity of the IMD images correlates with intratumor perfusion and microcirculation variability, providing a more precise depiction of tumor blood supply compared to routine enhanced CT scans (9–11). Nevertheless, the current analysis of IMD images is mainly based on a single iodine concentration value, which cannot fully reflect the heterogeneity of tumors or fully utilize the imaging features of IMD images. In parallel, histogram analysis presents a noninvasive and quantitative medical image analysis approach to assess tumor heterogeneity. This method involves the extraction and quantification of grayscale variations among pixels within an image, offering multiple quantitative parameters for a comprehensive tumor assessment (12, 13). This technology is widely used in the field of medical image processing and performs well in the diagnosis, differential diagnosis, and prognostic assessment of various tumors (12–15).

To date, no study has explored the potential of histogram analysis based on IDM images from DECT for preoperative differentiation of GS and GST (≤5 cm). Therefore, this study aimed to investigate the value of IMD histogram analysis in differentiating GS and GST (≤5 cm) before operation.

## Materials and methods

### Patient selection

This study was approved by the Ethical Review Board on Clinical Studies at our institution, and the requirement of informed consent was waived due to its retrospective nature. In total, 15 patients with GS and 30 patients with GST who underwent abdominal biphasic contrast-enhanced DECT between January 2015 and January 2023 were selected and included in this study based on the following inclusion criteria: (a) single tumor with a maximum diameter of less than 5 cm, (b) confirmed diagnosis of either GS or GST through postoperative pathological examination, and (c) abdominal biphasic contrast-enhanced DECT scans performed within 2 weeks before the surgery. The exclusion criteria were as follows: (a) any treatment before surgery, (b) poor image quality does not support the conduct of a histogram analysis, and (c) multiple gastric lesions. This study has been reported by the Standards for Reporting Diagnostic Accuracy Studies checklist.

### CT examination

All abdominal biphasic contrast-enhanced DECT scans were performed using the Discovery CT750 HD scanner (GE Healthcare, Waukesha, WI, USA) with the patients in the supine position. The patients were prepared by fasting for 6 to 8 h and were asked to drink 800–1,200 ml of warm water to distend the stomach at 10–15 min before the examination. Non-enhanced abdominal CT scan was performed using the conventional helical scan mode at a tube voltage of 120 kVp. The contrast-enhanced CT scans were performed by the DECT scanning mode using the following scan parameters: helical, rapid switch between tube voltages of 80 and 140 kVp in 0.5 ms; collimation thickness, 0.625 mm; tube current, 600 mA; rotation speed, 0.6 s; helical pitch, 0.983:1; slice thickness, 1.25 mm; and slice interval, 1.25 mm. Non-ionic contrast medium (iohexol, 300 mg iodine/mL) via antecubital venous access at a rate of 3.5–4.0 mL/s for a total of 80–100 mL (1.2 mL/kg of body weight) was injected to obtain biphasic contrast-enhanced images. The arterial phase (AP) scan began 20 s after the trigger attenuation threshold (100 HU) was reached above the level of the abdominal aorta, and the venous phase (VP) scan began 60 s after AP scanning. IMD images at the AP and VP were reconstructed at 1.25-mm image slice thickness and interval by basic material decomposition software on Advanced Workstation 4.7 (AW 4.7; GE Healthcare), respectively.

### Image analysis

Two radiologists, possessing 5 and 10 years of experience in abdominal imaging, analyzed all IMD images without knowing the patients’ clinical and pathologic information. The IMD images based on AP and VP were transferred to an offline workstation, and then the radiologists applied MaZda software (version 4.7, The Technical University of Lodz, Institute of Electronics, http://www.eletel.p.lodz.pl/mazda/) to conduct a histogram analysis, respectively. During the histogram analysis, a three-dimensional region of interest was outlined, and the MaZda software automatically extracted and selected nine histogram parameters, including mean, 1st, 10th, 50th, 90th, and 99th percentile of iodine concentration value (Perc.01, Perc.10, Perc.50, Perc.90, Perc.99), variance, skewness, and kurtosis. The histogram parameters were defined as follows: the mean reflects the average value of the pixel distribution, the *n*th percentile reflects the pixel value below *n*% of all pixel values, the variance reflects the degree of dispersion of the pixel distribution, the skewness reflects the asymmetry of the pixel distribution, and the kurtosis reflects the peakedness of the histogram. The X-axis of the histogram shows the pixel values, while the Y-axis is the cumulative frequency corresponding to the pixel values on the X-axis (12).

### Statistical analysis

Statistical analyses were performed using the MedCalc software (version 19.1, Mariakerke, Belgium) and SPSS software (version 21.0, IBM, Armonk, NY, USA). A value of *P* ≤ 0.05 was considered statistically significant. Inter-observer agreement was determined using the intraclass correlation coefficient (ICC) test (ICC < 0.40, poor agreement; 0.40 ≤ ICC < 0.60, moderate agreement; 0.60 ≤ ICC < 0.80, good agreement; and ICC ≥ 0.80, excellent agreement). Categorical variables were analyzed using the chi-square test. Continuous variables were compared using an independent *t*-test or Mann–Whitney *U*-test. The area under the curve (AUC), sensitivity, and specificity were obtained using receiver operating characteristic (ROC) curves to determine the diagnostic performance of significant histogram parameters. Bonferroni corrections were applied for multiple comparisons, and Delong’s test was used to compare the differences between AUCs.

## Results

### Patients’ clinical characteristics

The details of the clinical characteristics are presented in **Table 1**. The GS group comprised three male and 12 female patients (age range: 26–71 years; mean age: 53.33 ± 11.64 years), with a maximum tumor diameter of 3.24 ± 1.64 cm. The GST group (very low risk: 3, low risk: 11, intermediate risk: 10, and high risk: 6) comprised 10 male and 20 female patients (age range: 23–79 years; mean age: 55.40 ± 13.08 years), with a maximum tumor diameter of 3.46 ± 0.99 cm. There were no significant differences in sex, age, or maximum tumor diameter between the two groups (*P* = 0.561, *P* = 0.608, and *P* = 0.633, respectively).

**Table 1:** Patients’ characteristics.

Parameters	GS (*n* = 15)	GST (*n* = 30)	*P*
Sex			0.561
Males	3	10	
Females	12	20	
Age (years)	53.33 ± 11.64	55.40 ± 13.08	0.608
Tumor size (cm)	3.24 ± 1.64	3.46 ± 0.99	0.633

GS, gastric schwannoma; GST, gastric stromal tumor.

### Inter-observer agreement

The details of the ICC values of each IMD histogram parameter are listed in **Table 2**. Excellent inter-observer agreements were obtained for all IMD histogram parameters between the two groups of tumors (ICCs ranged from 0.852 to 0.988).

**Table 2:** Inter-observer agreement for IMD histogram parameters.

Parameters	Intraclass correlation coefficient	95% confidence interval
Mean	0.988	0.978–0.993
Perc.01	0.952	0.914–0.973
Perc.10	0.984	0.971–0.991
Perc.50	0.987	0.976–0.993
Perc.90	0.965	0.936–0.980
Perc.99	0.852	0.746–0.916
Variance	0.936	0.886–0.964
Skewness	0.966	0.940–0.981
Kurtosis	0.980	0.965–0.898

IMD, iodine-based material decomposition.

### Comparison of IMD histogram parameters between GS and GST

**Figure 1:**
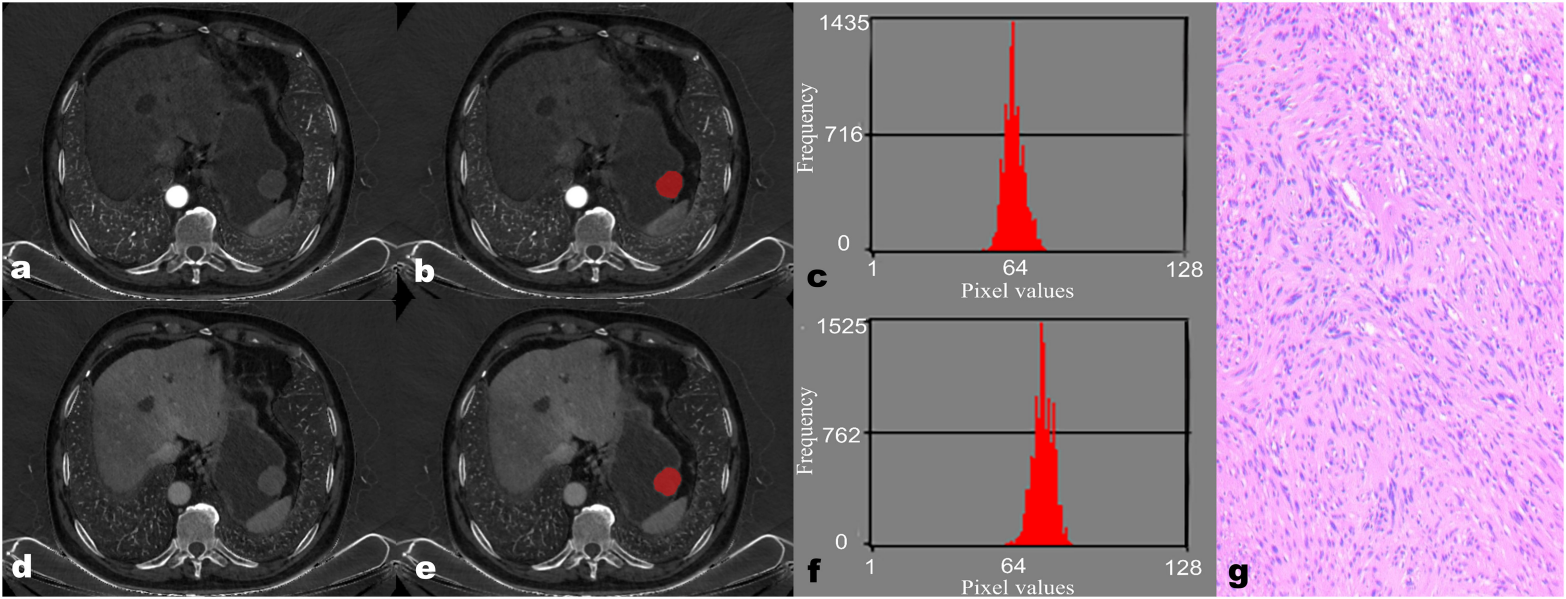
A 68-year-old male patient with gastric schwannoma (GS). The reconstructed axial iodine-based material decomposition (IMD) images of GS at the arterial phase **(A)** and venous phase **(D)** are shown. The tumor region of interest (ROI) was marked on the axial IMD image at the arterial phase **(B)** and venous phase **(E)**. Histogram of the ROI at the arterial phase **(C)** and venous phase **(F)**. Pathologically confirmed GS with spindle-shaped tumor cells arranged in a woven, fenestrated pattern **(G)** (hematoxylin and eosin, ×100).

**Figure 2:**
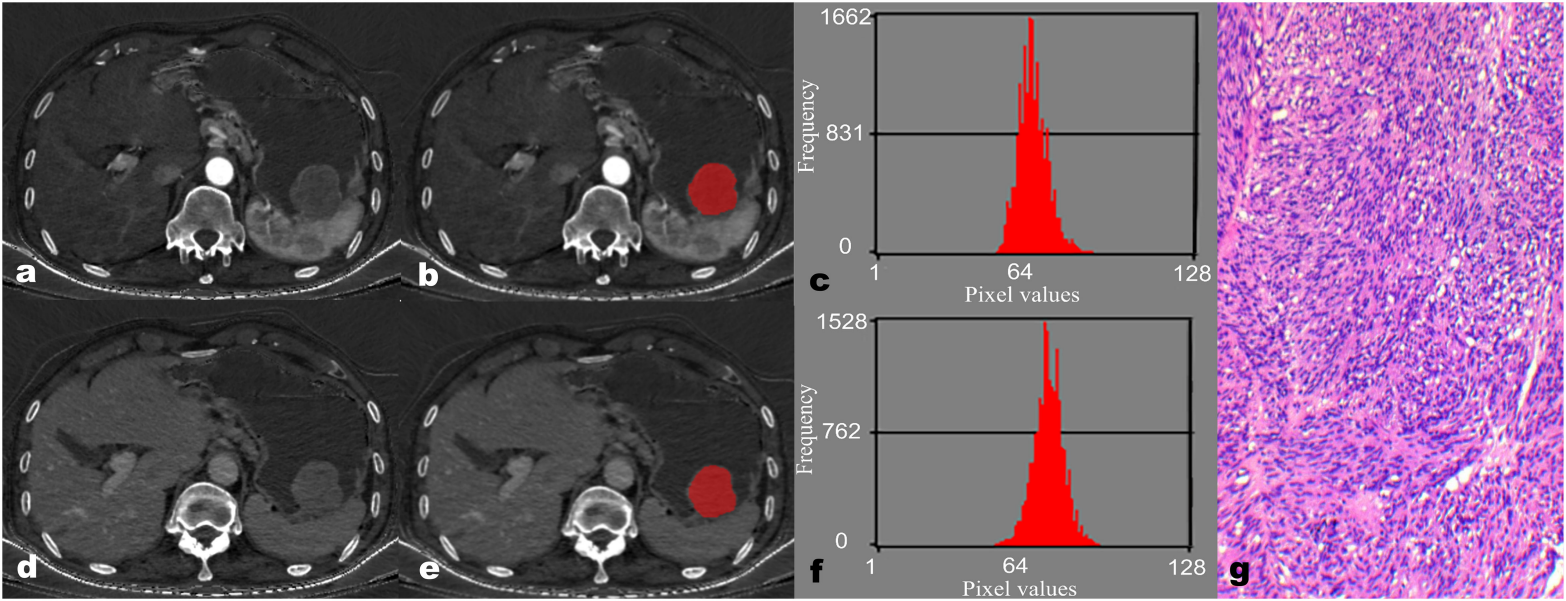
A 67-year-old male patient with gastric stromal tumor (GST). The reconstructed axial iodine-based material decomposition (IMD) images of GST at the arterial phase **(A)** and venous phase **(D)** are shown. The tumor region of interest (ROI) was marked on the axial IMD image at the arterial phase **(B)** and venous phase **(E)**. Histogram of the ROI at the arterial phase **(C)** and venous phase **(F)**. Pathologically confirmed GST with disorganized spindle cell arrangement, indistinct intercellular boundaries, and rare nuclear divisions **(G)** (hematoxylin and eosin, ×100).

Regarding the IMD histogram parameters of AP, the mean (58.02 ± 1.14 vs. 61.83 ± 5.33), Perc.50 (58.00 ± 1.00 vs. 62.00 ± 5.50), Perc.90 (63.00 ± 3.00 vs. 69.00 ± 2.50), Perc.99 (67.73 ± 2.63 vs. 75.63 ± 3.57), variance (17.50 ± 15.69 vs. 29.31 ± 20.03), and skewness (-0.06 ± 0.76 vs. 0.29 ± 0.57) of the GS group were significantly lower compared to the GST group (≤5 cm) (all *P* < 0.05). Similarly, regarding the IMD histogram parameters of VP, the Perc.90 (76.00 ± 2.00 vs. 77.50 ± 6.00), Perc.99 (81.00 ± 3.00 vs. 82.50 ± 7.00), and variance (22.76 ± 22.96 vs. 34.65 ± 23.18) of the GS group were significantly lower compared to the GST group (≤5 cm) (all *P* < 0.05). There was no statistical difference in kurtosis, Perc.01, and Perc.10 of IMD between the GS and GST groups (≤5 cm) (**Table 3**). Representative images of GS and GST (≤5 cm) are shown in **Figures 1**, **2**, respectively.

**Table 3:** Comparison of IMD histogram parameters between GS and GST (≤5 cm).

Parameters	Arterial phase			Venous phase		
	GS	GST	*P*	GS	GST	*P*
Mean	58.02 ± 1.14	61.83 ± 5.33	**0.017**	70.19 ± 1.50	70.95 ± 2.46	0.281
Perc.01	49.00 ± 9.00	50.50 ± 9.30	0.233	58.00 ± 15.00	55.00 ± 6.00	0.183
Perc.10	53.00 ± 2.00	56.00 ± 8.30	0.230	63.93 ± 1.87	63.23 ± 2.01	0.267
Perc.50	58.00 ± 1.00	62.00 ± 5.50	**0.023**	70.00 ± 2.00	71.00 ± 4.30	0.541
Perc.90	63.00 ± 3.00	69.00 ± 2.50	***P<*0.001**	76.00 ± 2.00	77.50 ± 6.00	**0.040**
Perc.99	67.73 ± 2.63	75.63 ± 3.57	***P<*0.001**	81.00 ± 3.00	82.50 ± 7.00	**0.004**
Variance	17.50 ± 15.69	29.31 ± 20.03	***P<*0.001**	22.76 ± 22.96	34.65 ± 23.18	**0.007**
Skewness	-0.06 ± 0.76	0.29 ± 0.57	***P<*0.001**	-0.38 ± 1.47	-0.27 ± 0.44	0.219
Kurtosis	0.33 ± 2.45	0.74 ± 1.22	0.219	0.56 ± 6.00	0.34 ± 1.19	0.904

IMD, iodine-based material decomposition; GS, gastric schwannoma; GST, gastric stromal tumor. Bold values represent P < 0.05.

### Diagnostic performance of IMD histogram parameters to differentiate GS and GST

**Figure 3:**
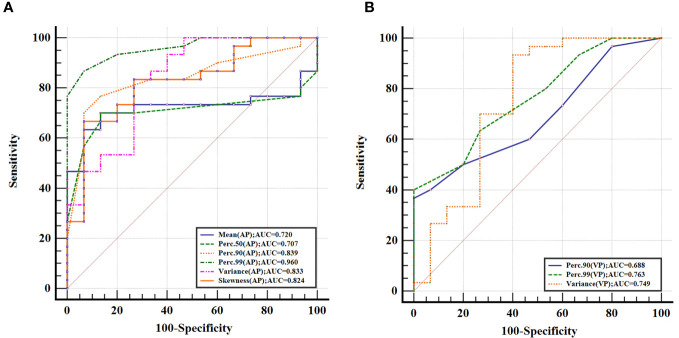
Receiver operating characteristic curves for histogram parameters based on iodine-based material decomposition images of dual-energy computed tomography in differentiating gastric schwannoma from gastric stromal tumor (≤5 cm) at the arterial phase **(A)** and venous phase **(B)**, with Perc.99 (arterial phase) generating the best diagnostic performance with the highest area under the receiver operating characteristic curve value (0.960; 95% CI, 0.855–0.996).

The ROC analysis showed that the Perc.99 (AP) generated the best diagnostic performance with the highest AUC (0.960; 95% CI, 0.855–0.996; *P* < 0.01), Using an optimal threshold of 71.00, this parameter achieved sensitivity and specificity of 86.67% and 93.33%, respectively, in differentiating GS between GST (≤5 cm). The analysis of ROC curves and the diagnostic performance of statistically significant IMD histogram parameters are shown in **Figure 3**; **Table 4**.

**Table 4:** Diagnostic performance of IMD histogram parameters to differentiate GS from GST (≤5 cm).

Parameters	AUC (95% CI)	Cutoff	Sensitivity (%)	Specificity (%)
Mean (AP)	0.720 (0.566, 0.843)	58.95	70.00	86.67
Perc.50 (AP)	0.707 (0.552, 0.833)	59.00	70.00	86.67
Perc.90 (AP)	0.839 (0.699, 0.931)	65.00	76.67	86.67
Perc.99 (AP)	0.960 (0.855, 0.996)	71.00	86.67	93.33
Variance (AP)	0.833 (0.692, 0.928)	24.63	83.33	73.33
Skewness (AP)	0.824 (0.692, 0.928)	0.23	66.67	93.33
Perc.90 (VP)	0.688 (0.532, 0.817)	79.00	36.67	100.00
Perc.99 (VP)	0.763 (0.613, 0.877)	85.00	40.00	100.00
Variance (VP)	0.749 (0.597, 0.866)	24.58	93.33	60.00

IMD, iodine-based material decomposition; CI, confidence intervals; AUC, area under the receiver operating characteristic curve; AP, arterial phase; VP, venous phase; GS, gastric schwannoma; GST, gastric stromal tumor.

## Discussion

This study focused on exploring the role of histogram analysis based on IMD images from DECT for the preoperative differentiation of GS and GST (≤5 cm). Our results suggest that IMD histogram analysis is an effective tool to differentiate between GS and GST (≤5 cm), with Perc.99 extracted from IMD images of AP being the most valuable potential parameter. To the best of our knowledge, this is the first study to use the IMD images for histogram analysis to distinguish between these two tumors.

IMD images represent a reconstructed image derived from DECT by basic material decomposition technology. This technique offers a more vivid depiction of iodine distribution within tissue structure when compared to the original image (8). Given that iodine is the primary constituent of iodine-containing contrast agents, quantifying the concentration of elemental iodine in tissue structures following the administration of such agents during contrast-enhanced scans can effectively portray the corresponding structures’ blood supply situation (16). The iodine concentration value serves as a prominent quantitative parameter for IMD analysis. Zhang LJ et al. (17) demonstrated a strong correlation between the iodine concentration value and CT perfusion parameters, making it a valid imaging marker for assessing tumor angiogenesis, metabolism, and blood supply. Malignant tumor cells exhibit heightened metabolism, active proliferation, and a robust tumor angiogenesis network, necessitating a significantly increased blood supply during the growth process (8, 18). Moreover, the incomplete endothelial cell structure in tumor angiogenesis leads to heightened vascular permeability. Consequently, iodine-containing contrast agents have an increased propensity to penetrate the tumor interior, resulting in a higher iodine concentration value (18, 19). Nevertheless, the iodine concentration value obtained from a single-level IMD image analysis of the lesion falls short of providing a comprehensive assessment of the tumor’s overall heterogeneity. Furthermore, it does not fully capture the deeper image information within the IMD images.

Therefore, as a rapidly emerging non-invasive medical image texture analysis tool, histogram analysis can provide a rich complement of image information, which could further extract image information and improve diagnostic accuracy (14, 15). Several studies have revealed that texture analysis based on IMD images of DECT can provide added value during tumor assessment (20, 21). Zeng F et al. (20) found that parameters obtained from texture analysis based on the IMD images from DECT are potential indices for predicting the axillary lymph node metastasis status in early-stage breast cancer. Chen Y et al. (21) constructed a radiomics nomogram based on IMD texture analysis to predict the peritoneal metastasis status of patients with gastric cancer, and the results showed that the model based on IMD images of DECT could effectively predict the peritoneal metastasis status of gastric cancer.

In this study, comprehensive histogram analysis was conducted on the entire tumor using biphasic IMD images obtained from DECT, and we observed excellent inter-reader agreement for all IMD histogram parameters. Specifically, among the IMD histogram parameters of AP, the mean, Perc.50, Perc.90, and Perc.99 of the GST group were notably higher when compared to the GS group, while among the IMD histogram parameters of VP, the Perc.90 and Perc.99 of the GS group were lower than those of the GST group. This divergence could be attributed to the active proliferation and robust metabolism of GST tumor cells, accompanied by an increased number of tumor blood vessels, resulting in a higher iodine concentration value in GST than in GS (18, 19). The mean and percentile of iodine concentration values essentially quantify the extent of iodine uptake by the tumor tissue. Liu J et al. (22) compared the difference in the iodine concentration value of AP and VP between 12 cases of GS and 20 cases of GST and found that the iodine concentration value in both phases of GST was higher than those of GS, which was consistent with the results of this study.

The variance mainly reflects the degree of change or dispersion of the grayscale of pixels in the image; a relatively larger variance usually implies a greater variation in the grayscale of pixels (12, 15). The results of this study showed significant differences in the variance of biphase IMD images between the two groups, and the variance of the GST group was higher than that of the GS group. One potential explanation is that it may be closely related to the different histopathological structural characteristics of the two groups of tumors. GS rarely shows necrosis and cystic degeneration; its structure is relatively homogeneous, and the contrast-enhanced scan shows a more uniform enhancement pattern (23). In contrast, GST has a high risk of necrosis and cystic degeneration compared to GS, regardless of its size, and the contrast-enhanced scans show a relatively heterogeneous enhancement pattern with a relatively larger variance (3, 24). Skewness represents asymmetry in the distribution of iodine concentration within the tumor. In this study, there was a significant difference in the skewness of the AP-IMD between GS and GST (≤5 cm), while there was no statistical difference in the skewness of the VP-IMD. The reason is hard to explain. One possible reason may be related to the different tumor vascular compositions between the two tumors (1, 2, 5).

This study also found that both IMD histogram analyses of biphasic DECT scans can provide several effective histogram parameters to distinguish between GS and GST. However, the number of significant histogram parameters at the AP was more than that at the VP, and the optimal parameter (Pec.99) to distinguish GS and GST (≤5 cm) originated from the AP, suggesting that the IMD images of AP may be more suitable for image analysis when differentiating between two types of tumor.

There were several limitations in our study. First, this is a single-center retrospective study, and an independent external validation set is necessary to validate our findings. Second, we did not separate risk grouping for GST due to the relatively small sample size. Finally, this study is a preliminary exploration of deep-level analysis on IMD images of GS and GST; only the first-order histogram parameters in texture features are analyzed, and the second-order parameters and high-order texture parameters need to be investigated for further research after expanding the sample size.

## Conclusion

In conclusion, the histogram analysis based on IMD images of DECT may be useful in the preoperative distinction of GS and GST (≤5 cm) and provides a quantitative reference for individual patient treatment planning.

## Data availability statement

The raw data supporting the conclusions of this article will be made available by the authors without undue reservation.

## Ethics statement

The studies involving humans were approved by the Ethical Review Board of Lanzhou University Second Hospital. The studies were conducted in accordance with the local legislation and institutional requirements. The ethics committee/institutional review board waived the requirement of a written informed consent for participation from the participants or the participants’ legal guardians/next of kin due to its retrospective nature.

## Author contributions

GW: conception and design, data collection, data analysis, drafting of manuscript, major revisions, and approval of submission on behalf of all authors. XL and JZ: data analysis, drafting of manuscript, major revisions, and article revision suggestions. All authors contributed to the article and approved the submitted version.
